# HIV-Infected Former Plasma Donors in Rural Central China: From Infection to Survival Outcomes, 1985–2008

**DOI:** 10.1371/journal.pone.0013737

**Published:** 2010-10-29

**Authors:** Zhihui Dou, Ray Y. Chen, Zhe Wang, Guoping Ji, Guoping Peng, Xiaochun Qiao, Jihua Fu, Xiangdong Meng, Marc Bulterys, Ye Ma, Yan Zhao, Ning Wang, Fujie Zhang

**Affiliations:** 1 National Center for AIDS/STD Control and Prevention, Chinese Center for Disease Control and Prevention, Beijing, China; 2 National Institute of Allergy and Infectious Diseases, U.S. National Institutes of Health, Beijing, China; 3 Henan Center for Disease Control and Prevention, Zhengzhou, China; 4 Anhui Center for Disease Control and Prevention, Hefei, China; 5 Hubei Center for Disease Control and Prevention, Wuhan, China; 6 Shanxi Center for Disease Control and Prevention, Taiyuan, China; 7 Shandong Center for Disease Control and Prevention, Jinan, China; 8 Jilin Center for Disease Control and Prevention, Changchun, China; 9 Global AIDS Program, U.S. Centers for Disease Control and Prevention, Beijing, China; 10 Beijing Ditan Hospital, Beijing, China; McGill University Health Center, Canada

## Abstract

**Background:**

The HIV epidemic among former plasma donors (FPDs) in rural Central China in the early-mid 1990s is likely the largest known HIV-infected cohort in the world related to commercial plasma donation but has never been fully described. The objectives of this study are to estimate the timing and geographic spread of HIV infection in this cohort and to demonstrate the impact of antiretroviral therapy on survival outcomes.

**Methodology/Principal Findings:**

HIV-infected FPDs were identified using the national HIV epidemiology and treatment databases. Locations of subjects were mapped. Dates of infection and survival were estimated using the midpoint date between initial-final plasma donation dates from 1985–2008 among those with plasma donation windows ≤2 years. Among 37084 FPDs in the two databases, 36110 were included. 95% were located in focal areas of Henan Province and adjacent areas of surrounding provinces. Midpoint year between initial-final plasma donation dates was 1994 among FPDs with known donation dates. Median survival from infection to AIDS was 11.8 years and, among those not treated, 1.6 years from AIDS to death. Among those on treatment, 71% were still alive after five years. Using Cox proportional hazard modeling, untreated AIDS patients were 4.9 times (95% confidence interval 4.6–5.2) more likely to die than those on treatment.

**Conclusions/Significance:**

The epidemic of HIV-infected FPD in China was not widespread throughout China but rather was centered in Henan Province and the adjacent areas of surrounding provinces. Even in these areas, infections were concentrated in focal locations. Overall, HIV infections in this cohort peaked in 1994, with median survival of 13.4 years from infection to death among those not treated. Among AIDS patients on treatment, 71% were still alive after five years.

## Introduction

In central China in the early-mid 1990s, plasma donation was promoted by collectors among poor, rural farmers as an easy way to supplement meager incomes. Some collectors established commercial plasma collection stations and, after collecting whole blood, removed the plasma and returned pooled red blood cells from blood type-matched donors so that donors could donate more often without becoming anemic [1], [2]. This unsanitary practice, in contrast to the Ministry of Health issued regulations on blood-borne pathogen precautions [3], resulted in untold numbers of human immunodeficiency virus (HIV) and hepatitis C virus (HCV) infections among the former plasma donors (FPDs)[4]–[8] until it was discontinued in 1996 [9]. The extent of the HIV epidemic among the FPDs was not recognized until they started developing the acquired immunodeficiency syndrome (AIDS) and dying in large numbers in the early 2000s. Mass HIV screenings in known FPD regions were conducted primarily in 2004 and tens of thousands of infected FPDs were identified [10], [11]. In response, the Chinese government established the National Free Antiretroviral Treatment Program (NFATP), initially started among the FPDs [12], [13]. The NFATP has now scaled up nationwide with analyses demonstrating increasing CD4+ cell counts and reduced mortality outcomes [14], [15].

The HIV-infected FPD cohort in China is unique because it is likely the largest known HIV-infected cohort in the world related to commercial plasma donation. Almost all HIV infections occurred over a relatively short period of time in the early-mid 1990s. The FPDs were primarily poor, rural farmers with almost no injection drug use or commercial sex work in their communities and, after at-risk spouses and newborn children were also infected, new infections were essentially eliminated. Because HIV infections occurred en masse, progression to AIDS and death also occurred around the same times. Small studies of HIV-infected FPDs have been published but no study has previously examined the entire cohort or estimated precisely when and where infections occurred [5]–[8], [16]–[18]. Identifying when and where infections occurred is essential to understanding the natural history and geographic spread of HIV infection in this cohort. In this analysis, we estimate the time and geographic spread of HIV infection among FPDs in China and demonstrate the impact of antiretroviral therapy on survival outcomes.

## Methods

### Ethics Statement

This analysis was approved by the institutional review board (IRB) of the National Center for AIDS/STD Control and Prevention (NCAIDS), China CDC. Per the IRB review, individual informed consent was waived because this analysis used currently existing data collected during the course of routine treatment and care and the data were reported in aggregate without the use of individual identifying information.

HIV infected individuals in this study were identified through an analysis of two national ongoing, prospective, observational databases. The first is an epidemiological database of all HIV-infected patients in China reported to the Chinese Center for Disease Control and Prevention (China CDC) through the national surveillance system, initially established in 1997. Former plasma donors still alive during the mass screenings around 2004 completed a questionnaire about their plasma donating activities from the early to mid-1990s. Data from FPDs who had already died were not captured. The second is a treatment database, previously described [19], of all subjects receiving free national treatment based on the national HIV treatment criteria (WHO stage III or IV disease, CD4+ cell count <200 cells/µL [increased to <350/µL in 2008], or total lymphocyte count <1200/µL) [20]. Antiretroviral treatment scaled up nationwide in 2003 with data prospectively included in the database since 2004. Information in the databases include demographics, suspected route of infection, date and method of HIV diagnosis, date of AIDS diagnosis, CD4+ cell counts pre- and post-treatment initiation, and date of death. In addition, the epidemiological database also captures self-reported dates of first and last plasma donations. When only a month was provided without a specific day, the 1^st^ of the month was used. When only a year was provided without a specific month or day, January 1 was used.

Only individuals infected by plasma donation were eligible for this analysis. Because the first case of HIV infection in China was identified in 1985 in a foreigner [21], FPD subjects were not thought to be infected with HIV in China before 1985. Plasma donations recorded before 1985 were not felt to be the source of HIV infection and subjects with this history were excluded. Subjects with other HIV risk factors and those missing key dates needed to calculate infection times and survival were also excluded. Those initiating treatment had 16 different signs or symptoms collected at baseline (yes/no). We included five that more typically represent immunosuppression (fever, diarrhea, dyspnea, oral candidiasis, and oral hairy leukoplakia) in the mortality risk factor analysis and did not include the less specific signs/symptoms, such as cough, headache, or visual changes. The most commonly used treatment regimens were nevirapine with zidovudine or stavudine and didanosine or lamivudine.

The date of HIV infection was estimated as the midpoint between the initial and final plasma donation dates. Only subjects with an initial-to-final plasma donation date range of ≤2 years were used to estimate infection times, due to concerns that intervals longer than two years may substantially bias survival estimates [22]. The baseline characteristics of these subjects were compared against subjects with initial-to-final dates longer than two years or with missing dates using the chi-squared test to determine if there were significant differences between these two groups. The geographic distributions of HIV-infected FPDs were plotted on a map (MapInfo Professional, version 6.5; Pitney Bowes Software Inc., Troy, New York) using county level data, with the midpoint of the ≤2 year cohort used to estimate the timing of HIV spread across different counties. Kaplan-Meier survival curves were used to estimate time from HIV infection to AIDS diagnosis (earliest date of AIDS-defining illness or CD4+ cell count <200/µL) and from AIDS diagnosis to death for the ≤2 year cohort. Surviving patients were censored on their date of last contact recorded in the databases or on 30-Aug-2008 and the log-rank test was used to compare survival curves. Cox proportional hazard modeling was used to assess risk factors for disease progression, from infection to AIDS and from AIDS to death. Covariates pre-determined to be clinically relevant were entered into multivariable models. Data were analyzed using SPSS version 17.0 (SPSS Inc., Chicago, Illinois) and SAS version 9.13 (SAS Institute Inc., Cary, NC). All hypothesis testing was two-sided with alpha = 0.05.

## Results

**Figure 1:**
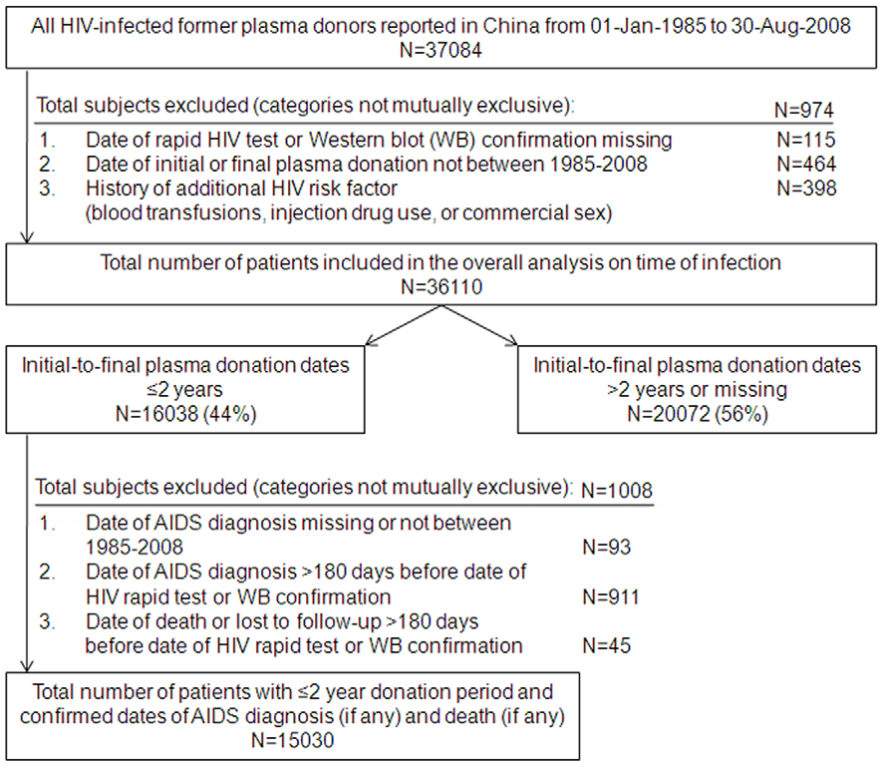
Selection criteria for HIV-infected former plasma donors (FPD) included in the analysis. Selection criteria for the 36110 HIV-infected former plasma donors (FPD) included in the overall analysis, the 16038 FPDs included in the estimation of the date of HIV infection, and the 15030 FPDs included in the survival analysis.

**Figure 2:**
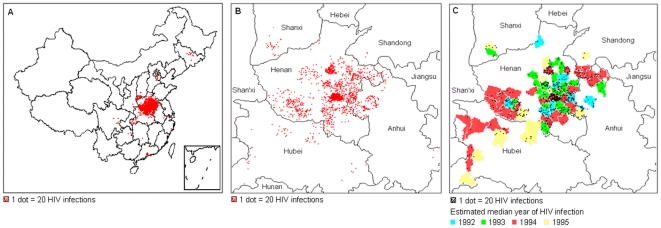
Distribution of HIV infection by region among former plasma donors (FPD) included in the analysis. Figure 2a: Map of the People's Republic of China showing the geographic distribution of all 36110 HIV-infected FPDs included in this analysis by location. Each dot represents 20 people. Figure 2b: Close-up map of Henan Province and the surrounding province showing the geographic distribution of all HIV-infected FPDs included in this analysis from this region. Each dot represents 20 people. Figure 2c: Close-up map of Henan Province and the surrounding province showing the geographic distribution of only the 16038 HIV-infected FPDs included in this analysis from this region with a ≤2 year plasma donation window. Estimated median peak year of HIV infection is indicated by county. Each dot represents 20 people.

Between the two national databases, 37084 individuals were identified as being HIV-infected by plasma donation, with 36110 subjects included in the overall analysis (Figure 1). These FPDs were predominantly male (58%), married (81%), educated to primary school (63%), and farmers (95%, Table 1). Median age (calculated) at infection was 29 years (interquartile range, 24–36). Almost 80% were from Henan Province. Initial and final plasma donation dates were recorded for 25960 (72%), with January recorded as the month in 48% and the first of the month as the date in 97%. Among those with recorded dates, 16038 (62%) donated plasma within a two year window and 9922 (38%) donated plasma for longer than 2 years. When baseline demographics of those donating within a two year period were compared to the remaining FPDs (>2 years and missing dates), all characteristics were statistically significantly different from each other due to the large sample size (Table 1). These differences, however, were not clinically meaningful except that slightly more of those donating within a two year period were from Henan Province. About 95% of all FPDs were concentrated in focal areas of Henan and the adjacent areas of surrounding provinces (Table 1, Figure 2a, 2b).

**Table 1:** Characteristics of the 36110 former plasma donors included in the analysis from 1985–2008, stratified by plasma donation window ≤2 years vs. >2 years.

Characteristic	Total	Interval between dates of first and last plasma donation		P-value
		0–2 years	>2 years or missing dates†	
	No. (%)	No. (%)	No. (%)	
Overall	36110	16038	20072	
Gender				
Male	20875 (57.8)	9137 (57.0)	11738 (58.5)	<0.001
Female	15235 (42.2)	6901 (43.0)	8334 (41.5)	
Estimated age at HIV infection (years)				
<18	—	765 (4.8)	—	—
18–44	—	14349 (90.4)	—	
≥45	—	767 (4.8)	—	
Missing		157		
Ethnicity				
Han	35243 (97.6)	15831 (98.7)	19412 (96.7)	<0.001
Other	867 (2.4)	207 (1.3)	660 (3.3)	
Marriage*				
Married/Live Together	28921 (81.2)	12868 (80.3)	16053 (82.0)	<0.001
Single/Divorced/Widowed	6680 (18.8)	3147 (19.7)	3533 (18.0)	
Missing	509	23	486	
Education*				
Primary school & below	22021 (63.0)	10138 (63.7)	11883 (62.5)	0.013
Middle school & above	12908 (37.0)	5765 (36.3)	7143 (37.5)	
Missing	1181	135	1046	
Occupation*				
Farmer	34163 (94.6)	15511 (96.7)	18652 (92.9)	<0.001
Others	1947 (5.4)	527 (3.3)	1420 (7.1)	
Province*				
Henan	28644 (79.3)	13489 (84.1)	15155 (75.5)	<0.001
Anhui	3189 (8.8)	1360 (8.5)	1829 (9.1)	
Hubei	1523 (4.2)	650 (4.1)	873 (4.3)	
Shanxi	724 (2.0)	206 (1.3)	518 (2.6)	
Shandong	198 (0.5)	91 (0.6)	107 (0.5)	
Other	1832 (5.1)	242 (1.5)	1590 (7.9)	
Estimated year of HIV infection				
≤1990	—	554 (3.5)	—	—
1991	—	1032 (6.4)	—	
1992	—	2649 (16.5)	—	
1993	—	3669 (22.9)	—	
1994	—	5174 (32.3)	—	
1995	—	2469 (15.4)	—	
1996	—	313 (2.0)	—	
≥1997	—	178 (1.1)	—	

† N = 9922 for those with interval >2 years and N = 10150 for those with missing dates.

*Data as of when the person was identified as HIV positive.

**Figure 3:**
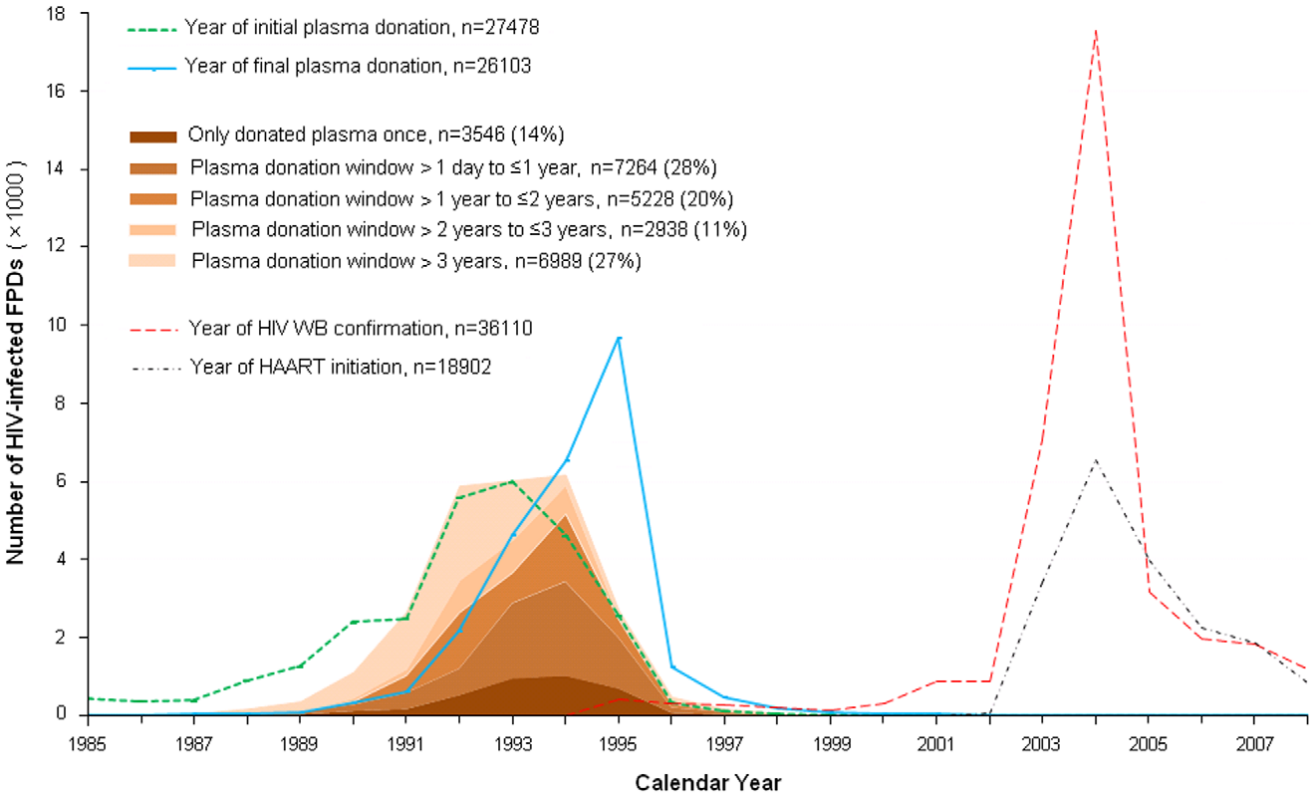
Distribution of the estimated time of HIV infection. Distribution of the estimated time of HIV infection among the subset of former plasma donors (FPD; N = 25960) with known initial and final plasma donation dates, using the midpoint of the donation window as the date of infection and stratified by the duration of the plasma donation window, from those who only donated once (first and last donation dates the same) to those who donated for more than three years. Dates of Western blot (WB) confirmation of HIV infection and initiating highly active antiretroviral therapy (HAART) are also shown.

Among the 25960 with known plasma donation dates, the peak midpoint year between initial and final donations was 1994 (Figure 3). There was a relatively gradual rise in initial donations before 1993, followed by a sharp drop in final plasma donations in 1996, when commercial plasma donation became illegal [9]. The vast majority were confirmed HIV positive by Western blot in 2004 due to mass screenings [10], [11], with HAART begun shortly thereafter for those still alive who met treatment eligibility criteria. Among the 16038 who donated plasma within a two year window, the estimated peak year of infection overall was also 1994 (32.3%, Table 1). When stratified by counties, however, HIV infections did not occur simultaneously across the provinces. In some counties in Henan and Northern Anhui, infections peaked as early as 1992 (Figure 2c).

**Figure 4:**
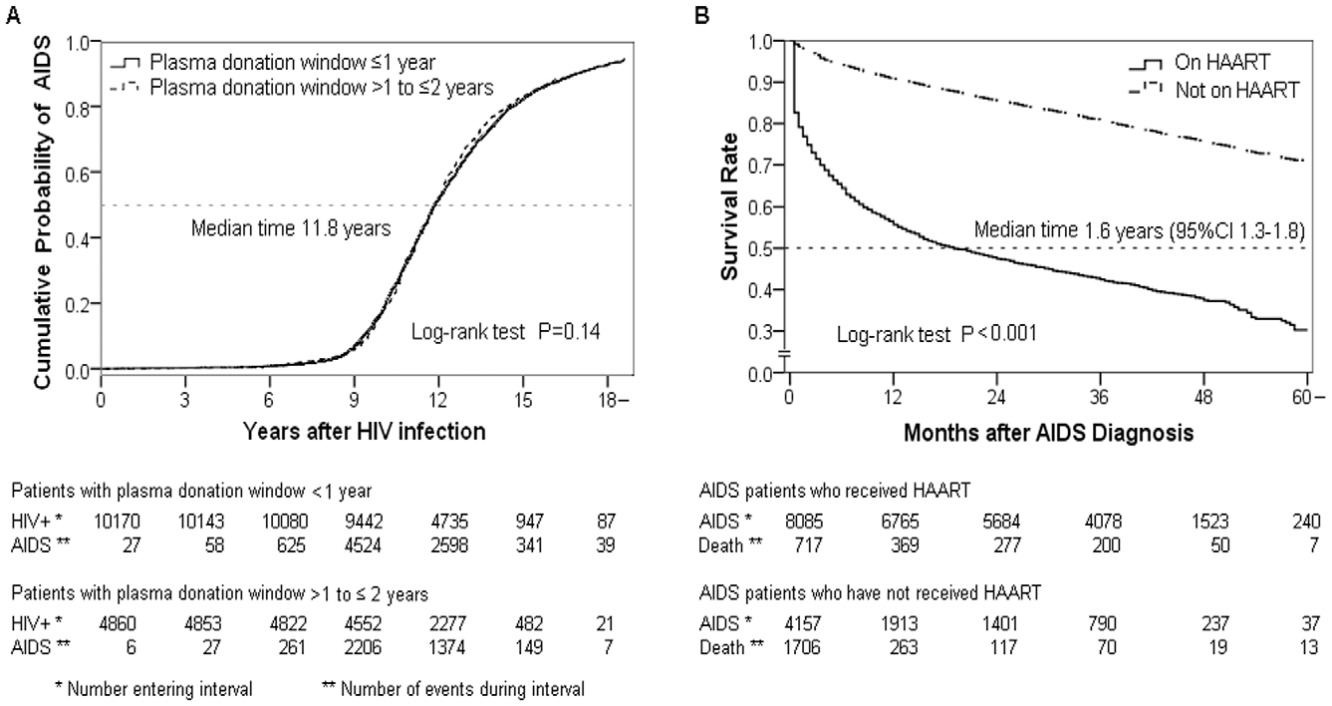
Survival from HIV infection to AIDS and from AIDS to death. Kaplan-Meier plots showing survival from HIV infection to AIDS in former plasma donors with plasma donation window ≤2 years (Figure 4a, N = 15030) and from AIDS to death among the same cohort who were diagnosed with AIDS (N = 12242), stratified by whether or not on highly active antiretroviral therapy (HAART; Figure 4b).

Among those with a plasma donation window of ≤2 years, an additional 1008 FPDs were excluded with missing or unconfirmed dates for the survival analysis (Figure 1). Among the remaining 15030 subjects, there was a median 11.8 years from HIV infection to AIDS, with no difference noted between those with a plasma donation window of 0–1 year versus >1 to 2 years (Figure 4a). Among those not receiving HAART, 45% died after 1 year with a median 1.6 years from AIDS to death, for a mortality rate of 34.3/100 person-years (Figure 4b). In contrast, mortality among those receiving HAART at one year was only 7% with 71% still surviving at five years (overall mortality rate 6.7/100 person-years). Risk factors associated with HIV disease progression from infection to AIDS and from AIDS to death were examined (Table S1). From HIV infection to AIDS, only three factors could be analyzed because the data used (gender and ethnicity) were collected at the time of HIV diagnosis. The age at infection was estimated as described above. In the adjusted analysis, both gender and ethnicity were of borderline significance while increasing age at time of infection was associated with a significantly greater risk of disease progression. Compared to <18 year olds, 18–44 year olds were 1.3 fold (95% confidence interval [CI] 1.2–1.5) more likely to develop AIDS and ≥45 years olds were 1.6 fold (95% CI 1.4–1.8) more likely. In the overall adjusted analysis from AIDS to death, treatment with HAART was by far the most important risk factor, with those not receiving HAART being 4.9 fold (95% CI 4.6–5.2) more likely to die. Among AIDS patients who received HAART, factors significantly associated with death included baseline CD4+ cell count <50/µL (adjusted hazard ratio [AHR] 3.1, 95% CI 2.1–4.7), age ≥60 years (AHR 2.8, 95% CI 1.4–5.6), baseline hemoglobin <8 g/dL (AHR 2.4, 95% CI 1.6–3.5), having 4–5 baseline signs or symptoms (AHR 1.7, 95% CI 1.2–2.5), and being male (AHR 1.4, 95% CI 1.1–1.7).

## Discussion

The specific circumstances surrounding when and where the unique cohort of FPDs in China became infected with HIV have never before been fully analyzed. This analysis shows that about 95% of HIV-infected FPDs were located in focal areas of Henan and adjacent areas of surrounding provinces. Using a subset of HIV-infected FPDs with known plasma donation dates within a two year window to represent the entire cohort, we estimated that the overall peak year of HIV infections was 1994 (Table 1) but as early as 1992 in some counties in Henan and Northern Anhui (Figure 2c). The median time from HIV infection to AIDS was 11.8 years and, among those not on HAART, from AIDS to death was 1.6 years (Figure 4). The major risk factor for development of AIDS was being older at time of HIV infection and for death was not receiving HAART (Table S1). Among those who received HAART, additional risk factors for death included baseline CD4 cell count <50/µL, age ≥60 years at AIDS diagnosis, baseline hemoglobin <8 g/dL, and having 4–5 baseline signs and symptoms at treatment initiation.

The concentration of this HIV epidemic in Henan Province is striking and the reasons for why this occurred are complex. The primary source of plasma used by industry is from developing countries and plasma donors in poor countries are less likely to be injection drug users (IDUs) [23]. Thus, it was felt that these mostly rural and poor populations, such as in Henan Province, were “safer,” without many risk factors for blood-transmitted infections such as HBV, much less HIV. In the early 1990s, Henan permitted paid plasma donation at commercial plasma collection stations, which was seen in rural areas as a relatively easy means of income supplementation. Donating plasma quickly became popular as people felt it was less harmful than donating whole blood since they received red blood cells back and could replace plasma easily by drinking more fluids. Once the government realized that HIV had entered the plasma pool, they acted quickly to shut down the collection stations. By that time, however, tens of thousands had already become infected. This FPD epidemic was not caused by injection drug use as demonstrated by distinct molecular epidemiology patterns among FPDs and IDUs in China [24]. Previous studies focused on determining HIV prevalence and risk factors among FPD communities and found prevalences ranging from 7.2–25.9% among those who previously donated plasma [7], [8], [16], [17]. Hepatitis B virus (HBV) infections were uncommon in FPDs because those infected were screened out before donating. Hepatitis C virus infection testing was not yet available at that time and thus co-infection rates with HIV have been reported to be quite high [5], [6], [18], [25], [26]. In our study, HIV-infected FPDs were not homogenously distributed across Henan but were located in focal areas (Figure 2c). It is likely that, if we were able to use village (instead of county) level data, the focal areas would be even more distinctly defined. These focal areas are hypothesized to be where previous commercial plasma collection stations were located. The majority of infections were in central Henan, near the border with Anhui, and HIV appears to have entered certain counties in Henan and Northern Anhui as early as 1992. Infections peaked in the surrounding counties in subsequent years, suggesting that commercial plasma collection stations branched out to those areas. Alternatively, it is possible that commercial plasma collection stations were already widespread but that HIV first entered the ones that adhered the least to blood-borne pathogen precautions

A number of studies have analyzed the natural history of HIV infection and estimated the time from seroconversion to death, ranging from 7.5–12.6 years in different populations [27]–[33]. Among our FPDs, median time from seroconversion to death was slightly longer at 13.1 years, due to the survival bias in our cohort. Mass HIV screening surveys were done in 2004–2005 [10], [11], when most of the baseline demographics data in the databases were collected. This was likely at least ten years after HIV infection occurred for most. Missing from the screening surveys, therefore, were those who had already developed AIDS and died, with shorter times to death, or those with typical times to death but were infected earlier. The bias from shorter times to death may be large because many people donated repeatedly and higher HIV inoculum received has been correlated with faster progression to AIDS [34]. Thus the true median time from infection to death is shorter than our estimate of 13.4 years. The bias from those infected earlier affects our estimate of the date of HIV infection, as those infected earlier (and therefore died earlier) were not included. Thus, the true peak time of infection is possibly before 1994. Due to insufficient HIV infection and mortality data from the 1990s to the early 2000s, the extent of these survival biases on our results is uncertain.

The impact of HAART in significantly reducing mortality among AIDS patients in this study (Figure 4) validates our previous analysis of decreased mortality among a subset of 4093 FPD AIDS patients [14]. In that study, we demonstrated that mortality declined from 30.2/100 person-years pre-treatment to 4.6/100 person-years post-treatment. Our current results are also consistent with our analysis of the five year outcomes of the NFATP, which included 48,785 patients with FPDs comprising over 50% [15]. We demonstrated that mortality among treated AIDS patients dropped from 22.6/100 person-years at treatment initiation to 4–5/100 person-years across five years of treatment. The results of this study, with a Kaplan-Meier analysis showing continued survival of 71% of treated FPD AIDS patients at five years, further confirms the positive effect of the NFATP in reducing long-term mortality.

There are a few limitations associated with this analysis, in addition to the survival bias discussed above. First, because baseline demographics data, including initial and final plasma donation dates, were collected primarily in 2004–2005, there may be misclassification of donation durations regarding the plasma donation dates (within two years versus greater than two years or unknown), which occurred roughly 10 years earlier. However, this misclassification error is felt to have a random effect, not systematically altering the donation dates to be earlier or later. Furthermore, our estimate of 13.4 years survival from infection to death is consistent with previous natural history estimates, accounting for the survival bias in our cohort. Second, we only use the 44% of FPDs with up to a two year donation window to represent all FPDs when estimating time of infection and survival. However, the use of a two year window has been validated by other studies [22]. Furthermore, there were no clinically significant differences between the baseline demographics and regional distribution (Table 1) or temporal distribution (Figure 3) between our cohort and those with larger or unknown donation windows. Third, with so many infections concentrated over a relatively short period of time (few years), the concentration of HIV in the pooled plasma of donors may have been quite high, especially during the peak infection years. Infections may have occurred early during the plasma donation period rather than at the midpoint. However, limiting the plasma donation period to one year did not change the results (Figure 4a). Finally, there is likely bias due to residual confounding. Several potentially confounding factors could not be included in the Cox proportional hazards regression analysis of HIV disease progression (Table S1) because the data were collected at the time of HIV diagnosis, roughly ten years after HIV infection first occurred. Only gender, age (calculated), and race/ethnicity would have been unchanged at the time of infection and therefore could be included in the analysis. Other relevant factors or potential confounders, such as details related to the plasma donation events, were not known.

In conclusion, this analysis demonstrates the devastating impact that not adhering strictly to blood-borne pathogen precautions can have, infecting tens of thousands of people with HIV across central China. Infections peaked in 1994, with 95% of HIV-infected FPDs concentrated in focal areas of Henan and the adjacent areas of surrounding provinces. These results are generalizable to all FPDs in China, given the above limitations, and have implications for those still alive. Studies have shown high rates of HCV co-infection in this population [5], [6], and HCV was likely acquired at or around the time of HIV infection. With the date of HIV infection now estimated, the natural history of HCV infection can be studied and strategies to deal with chronic HCV infection can be examined. Future large-scale blood product practices in resource-limited settings must be certain to adhere strictly to international blood-borne pathogen precautions to prevent repeat occurrences of such a public health disaster.

## Supporting Information

Table S1 (0.12 MB DOC)
