# Supplementary material for: HIV-Infected Former Plasma Donors in Rural Central China: From Infection to Survival Outcomes, 1985–2008
Source: PLoS One. 2010 Oct 29;5(10):e13737. doi: 10.1371/journal.pone.0013737 (PMC2966407; doi:10.1371/journal.pone.0013737)
Supplement: Table S1 — (0.12 MB DOC) [file pone.0013737.s001.doc]

Table 2: Cox proportional hazard regression analysis of factors associated with HIV disease progression, from HIV infection to AIDS and from AIDS to death, among former plasma donors in China, 1985-2008.

| Factor | No. | Total | | No. | On HAART | | No. | Not on HAART | |
| --- | --- | --- | --- | --- | --- | --- | --- | --- | --- |
|  |  | Unadjusted | Adjusted |  | Unadjusted | Adjusted |  | Unadjusted | Adjusted |
|  |  | HR (95% CI) | HR (95% CI) |  | HR (95% CI) | HR (95% CI) |  | HR (95% CI) | HR (95% CI) |
| **From HIV Infection to AIDS** | 15030 |  |  |  |  |  |  |  |  |
| Gender |  |  |  | — | — | — | — | — | — |
| Female | 6417 | 1.0 | 1.0 |  |  |  |  |  |  |
| Male | 8613 | 0.9 (0.9-1.0) | 1.0 (0.9-1.0) |  |  |  |  |  |  |
| Estimated age at HIV infection, years |  |  |  | — | — | — | — | — | — |
| <18 | 720 | 1.0 | 1.0 |  |  |  |  |  |  |
| 18-44 | 13586 | 1.3 (1.2-1.4) | 1.3 (1.2-1.5) |  |  |  |  |  |  |
| ≥45 | 716 | 1.8 (1.6-2.0) | 1.6 (1.4-1.8) |  |  |  |  |  |  |
| Race |  |  |  | — | — | — | — | — | — |
| Han | 14832 | 1.0 | 1.0 |  |  |  |  |  |  |
| Other | 132 | 0.8 (0.7-1.0) | 1.2 (1.0-1.5) |  |  |  |  |  |  |
| **From AIDS to Death** | 12242 |  |  | 8085 |  |  | 4157 |  |  |
| Sex |  |  |  |  |  |  |  |  |  |
| Female | 5331 | 1.0 | 1.0 | 3691 | 1.0 | 1.0 | 1680 | 1.0 | 1.0 |
| Male | 6911 | 1.5 (1.4-1.6) | 1.4 (1.3-1.5) | 4394 | 1.5 (1.4-1.7) | 1.5 (1.2-1.7) | 2624 | 1.1 (1.1-1.4) | 1.2 (1.1-1.3) |
| Age at AIDS diagnosis, years |  |  |  |  |  |  |  |  |  |
| <30 | 729 | 1.0 | 1.0 | 441 | 1.0 | 1.0 | 295 | 1.0 | 1.0 |
| 30-44 | 7557 | 0.9 (0.8-1.1) | 1.0 (0.9-1.2) | 5014 | 1.0 (0.8-1.3) | 1.4 (1.0-2.1) | 2623 | 0.9 (0.8-1.0) | 0.9 (0.8-1.1) |
| 45-59 | 3649 | 0.9 (0.8-1.1) | 1.1 (1.0-1.3) | 2464 | 1.2 (0.9-1.4) | 1.7 (1.1-2.5) | 1234 | 0.9 (0.8-1.1) | 1.0 (0.9-1.2) |
| ≥60 | 299 | 1.7 (1.3-2.0) | 1.6 (1.3-2.0) | 166 | 2.0 (1.4-2.9) | 3.6 (2.1-6.2) | 144 | 1.3 (1.0-1.6) | 1.3 (1.02-1.7) |
| Race |  |  |  |  |  |  |  |  |  |
| Han | 12090 | 1.0 | 1.0 | 8008 | 1.0 | 1.0 | 4229 | 1.0 | 1.0 |
| Other | 105 | 2.4 (1.8-3.1) | 1.6 (1.3-2.1) | 45 | 1.6 (0.9-2.9) | 1.1 (0.4-2.6) | 60 | 1.8 (1.4-2.4) | 1.7 (1.3-2.3) |
| Marital status |  |  |  |  |  |  |  |  |  |
| Married/Live together | 9852 | 1.0 | 1.0 | 6623 | 1.0 | 1.0 | 3371 | 1.0 | 1.0 |
| Single/Divorced/Widowed | 2374 | 1.3 (1.2-1.4) | 1.1 (1.0-1.2) | 1454 | 1.2 (1.1-1.4) | 1.2 (0.9-1.4) | 927 | 1.2 (1.1-1.3) | 1.1 (0.9-1.2) |
| Education |  |  |  |  |  |  |  |  |  |
| Primary school & below | 7834 | 1.0 | 1.0 | 5171 | 1.0 | 1.0 | 2775 | 1.0 | 1.0 |
| Middle school & above | 4318 | 0.9 (0.9-1.1) | 0.9 (0.9-1.0) | 2862 | 1.0 (0.9-1.1) | 1.1 (0.9-1.3) | 1490 | 0.9 (0.8-1.0) | 0.9 (0.8-1.0) |
| Occupation |  |  |  |  |  |  |  |  |  |
| Farmer | 11877 | 1.0 | 1.0 | 7858 | 1.0 | 1.0 | 4162 | 1.0 | 1.0 |
| Other | 360 | 1.5 (1.3-1.8) | 1.7 (1.5-2.0) | 225 | 1.0 (0.7-1.3) | 0.8 (0.5-1.3) | 139 | 2.1 (1.8-2.6) | 2.1 (1.8-2.6) |
| Treated with HAART |  |  |  | — | — | — | — | — | — |
| Yes | 7938 | 1.0 | 1.0 |  |  |  |  |  |  |
| No | 4304 | 5.1 (4.7-5.4) | 4.9 (4.6-5.2) |  |  |  |  |  |  |
| Last pre-treatment CD4 cell count (within 365 days), cells/μL | — | — | — |  |  |  | — | — | — |
| ≥350 |  |  |  | 581 | 1.0 | 1.0 |  |  |  |
| 200-349 |  |  |  | 1071 | 1.4 (1.0-1.9) | 1.3 (0.9-2.0) |  |  |  |
| 50-199 |  |  |  | 2534 | 2.5 (1.9-3.5) | 2.6 (1.8-3.8) |  |  |  |
| <50 |  |  |  | 846 | 6.5 (4.8-8.8) | 5.9 (4.0-8.7) |  |  |  |
| Hemoglobin at HAART initiation, g/dL | — | — | — |  |  |  | — | — | — |
| ≥8 |  |  |  | 6428 | 1.0 | 1.0 |  |  |  |
| <8 |  |  |  | 221 | 1.8 (1.4-2.3) | 2.1 (1.5-2.9) |  |  |  |
| ALT at HAART initiation, U/L | — | — | — |  |  |  | — | — | — |
| ≤100 |  |  |  | 5779 | 1.0 | 1.0 |  |  |  |
| >100 |  |  |  | 144 | 1.3 (0.9-1.8) | 1.3 (0.9-2.1) |  |  |  |
| Fever* | -- | — | — |  |  |  | — | — | — |
| Yes |  |  |  | 5494 | 1.5 (1.3-1.7) | 1.4 (1.2-1.7) |  |  |  |
| No |  |  |  | 2589 | 1.0 | 1.0 |  |  |  |
| Diarrhea* | -- | -- | -- |  |  |  | -- | -- | -- |
| Yes |  |  |  | 3489 | 1.2 (1.1-1.3) | 1.2 (1.0-1.4) |  |  |  |
| No |  |  |  | 4591 | 1.0 | 1.0 |  |  |  |
| Dyspnea* | -- | -- | -- |  |  |  | -- | -- | -- |
| Yes |  |  |  | 1084 | 2.4 (2.2-2.7) | 1.6 (1.4-2.0) |  |  |  |
| No |  |  |  | 6993 | 1.0 | 1.0 |  |  |  |
| Thrush* | -- | -- | -- |  |  |  | -- | -- | -- |
| Yes |  |  |  | 1459 | 1.5 (1.3-1.7) | 1.1 (0.9-1.4) |  |  |  |
| No |  |  |  | 6620 | 1.0 | 1.0 |  |  |  |
| Oral hairy leukoplakia* | -- | -- | -- |  |  |  | -- | -- | -- |
| Yes |  |  |  | 643 | 1.6 (1.4-1.9) | 1.0 (0.8-1.3) |  |  |  |
| No |  |  |  | 7436 | 1.0 | 1.0 |  |  |  |

Note: HAART=highly active antiretroviral therapy; HR=hazard ratio; CI=confidence interval; * Signs/symptoms present or absent at baseline treatment initiation
